# 2-Bromopalmitate-Induced Intestinal Flora Changes and Testicular Dysfunction in Mice

**DOI:** 10.3390/ijms252111415

**Published:** 2024-10-24

**Authors:** Yuxuan Ma, Yining Chen, Long Li, Zifang Wu, Heran Cao, Chao Zhu, Qimin Liu, Yang Wang, Shaoxian Chen, Yuyan Liu, Wuzi Dong

**Affiliations:** College of Animal Science and Technology, Northwest A&F University, No. 22 Xinong Road, Yangling 712100, China; mayuxuanrl@126.com (Y.M.); yining.chen.cyn@gmail.com (Y.C.); 2021060188@nwafu.edu.cn (L.L.); zifang_wu@126.com (Z.W.); caoheran@nwafu.edu.cn (H.C.); chaozhu5739@163.com (C.Z.); liuqimin618@163.com (Q.L.); wy3356145351@163.com (Y.W.); 15717875330@163.com (S.C.); 13683402668@163.com (Y.L.)

**Keywords:** 2-bromopalmitate, intestinal flora, testicular dysfunction

## Abstract

2-Bromopalmitate (2-BP) is a palmitoylation inhibitor that can prevent the binding of palmitic acid to proteins, thereby exhibiting significant effects in promoting inflammation and regulating the immune system. However, limited research has been conducted regarding the direct effects of 2-BP on the animal organism. Therefore, we probed mice injected with 2-BP for altered expression of inflammatory genes, with a focus on demonstrating changes in the intestinal flora as well as damage to the reproductive system. Our findings indicate that 2-BP can induce substantial inflammatory responses in visceral organs and cause testicular dysfunction. The key changes in the gut microbiota were characterized by an abundance of *Firmicutes*, *Clostridiales*, *Rikenellaceae_RC9_gut_group*, *Desulfovibrio*, *Muribaculaceae*, and *Alistipes*, and their metabolism has been intricately linked to visceral inflammation. Overall, the findings of this study provide a sound scientific basis for understanding the impact of high doses of 2-BP in mammals, while also offering crucial support for the development of preclinical models to suppress palmitoylation.

## 1. Introduction

Post-translational modifications (PTMs) refer to chemical alterations capable of dynamically affecting the structure, function, and localization of a protein, thereby effectively modulating cellular processes [1]. Perturbations in the regulation of aberrant PTM conformations within cells can disrupt the activities of enzymes, ultimately leading to the onset of various diseases [2]. Protein palmitoylation is an important post-translational modification [3,4]. It has been found to be important for controlling protein trafficking and organelle localization and function [5]. Distinguished from other protein modifications, its process is reversible [6,7], favoring the dynamic regulation of proteins [8]. It has been shown that palmitoylation can affect inflammatory pathways and plays an important role in male reproduction [9,10,11]. The palmitoylated protein ZDHHC19 has been shown to be required in the process of spermatogenesis [12]. In addition, previous studies from our laboratory have shown that palmitoylation of the GLB1L4 protein is required to maintain sperm function in the rat epididymis [13]. Simultaneously, the palmitoylation of proteins is of great significance for providing energy for sperm motility, and once the balance of protein palmitoylation in the body is disrupted and the level of palmitic acid is increased, it may lead to damage to the male reproductive system [14]. If certain receptors’ palmitoylation sites are mutated, their corresponding effector proteins will lose their regulatory ability, thus causing damage to sperm production [15].

2-Bromopalmitate (2-BP) belongs to the class of non-metabolizable palmitate analogs and it can effectively block the incorporation of palmitate into proteins [16]. The effectiveness of this compound as a potent inhibitor of protein palmitoylation has been validated through experiments with numerous palmitoylated proteins [17,18,19,20]. 2-BP is used medically to treat neuropathic pain caused by various factors [21,22,23], as well as to inhibit the growth of tumor cells [24]. In addition, 2-BP has an inhibitory effect on the catabolic utilization of fatty acids [25]. Presently, the relevant research on 2-BP predominantly centers on rats, and studies have indicated that it exhibits a certain degree of toxicity to both male and female individuals as well as embryos [26,27,28]. Nevertheless, there is still no definite research on the impact of 2-BP on the male reproductive system and internal organs of mice. In addition, although we referred to the concentrations commonly used in rat experiments, a dose of 200 mg/kg would cause the death of mice after intraperitoneal injection.

In animals, most of the ingested fatty acids need gut flora for breakdown and utilization [29,30]. And bacteria are very important to animals; they live symbiotically in their intestines and play a major role in digestion and immune function [31,32]. At the same time, palmitoylation has a crucial role in bacterial colonization and survival in the organism [33,34]. In addition, the gut microbiota can effectively decompose and utilize palmitic acid, reducing the level of palmitamide in the blood, and this may have a certain impact on mice in which palmitoylation is inhibited after 2-BP injection [35,36]. Changes in gut flora often result from changes in the animal’s diet [37], such as high-fat diets or alcohol consumption [38,39]. In contrast, in this study, injected 2-BP caused changes in the gut flora, which is a very novel finding. Studies have shown that palmitoylated proteins can have a significant impact on the intestinal microbiota [40]. Once the palmitoylated proteins are imbalanced, the intestinal microbial structure of animals will change [41].

Therefore, in the present study, we investigated the inflammatory indexes of the internal organs, the composition of the intestinal flora, and the reproductive ability of male mice after 2-BP injection. We also analyzed the effect of 2-BP on the overall physiology of mice, in conjunction with alterations in intestinal flora, exploring the influence of palmitoylation inhibitors on the gut microbiota and whether the changes in the gut microbiota can mitigate the adverse reactions resulting from the inhibition of palmitoylation in the organism.

## 2. Result

### 2.1. Effects on Viscera

After the mice were weighed, they were euthanized via cervical dislocation. Subsequently, their hearts, livers, spleens, lungs, and kidneys were extracted and weighed for calculating their proportions in the overall body weight. However, the results demonstrated that there was no significant difference among the various groups of mice (Figure S1). Histological examination of HE-stained liver tissue sections revealed that the livers of mice in the 50-36d group exhibited moderate focal steatosis (Figure S2). The renal tubular epithelium in mice from the 50-10d group experienced vesicular degeneration. In addition, the lumen of renal tubules in the 100-36d group was dilated, with proliferation of the glomerular capillaries (Figure S3). The expression profiles of inflammatory factors revealed that in the liver, the expression levels of *Tnf-α* and IL10 in the 50-10d group as well as *Tnf-α* in the 100-36d group were relatively elevated, and the expression level of Il6 in the 50-36d group was comparatively high. However, other inflammatory factors did not exhibit an upregulated phenomenon (Figure S4A,D). In contrast to the liver, after the administration of a dose of 100 mg/kg, the expression levels of inflammatory factors in the kidneys of mice all showed more prominent increases (*Il1-α*, *Tnf-α*, *Il6* in the 100-10d group and *Il1-α*, *Tnf*, *Il10* in the 100-36d group) (Figure S4B,E). In the spleen, 2-BP injection elicited a more long-lasting response. On the 36th day, the expression levels of inflammatory factors in the mice spleen were significantly enhanced, while on the 10th day, only the expression levels of *Il1-α* and *Il6* were relatively high (Figure S4C,F). Consequently, the inflammatory response induced by 2-BP was more pronounced in the kidney and spleen and had a greater long-term impact on the spleen.

### 2.2. Effects on Intestinal Microorganisms in Mice

#### 2.2.1. Analysis of Intestinal Flora Diversity

There were no significant differences in alpha diversity (Figure S5). Principal Coordinate Analysis (PCoA) and Non-Metric Multidimensional Scaling (NMDS) based on Bray–Curtis distance were employed to assess the beta diversity of the gut microbiota in the mice treated with varying concentrations of 2-BP at the different time points. The results showed that under the three algorithms, the sample points of the 50-10d group and the 100-10d group had different degrees of interweaving with the confidence circles of the control group (Figure S6A–C), so their representative microbial communities were similar. Although in NMDS, the sample points of 100-36d had a certain overlap with the control group; under the other two algorithms, there was a significant separation from the confidence circle of the control group (Figure S6D–F). Thus, the treatment of 2-BP at 100 mg/kg can significantly change the structure of the intestinal microbiota of mice, and this change requires a certain amount of time. In the short term, the change in the intestinal microbiota is not significant.

#### 2.2.2. Intestinal Flora Composition Difference

**Figure 1:**
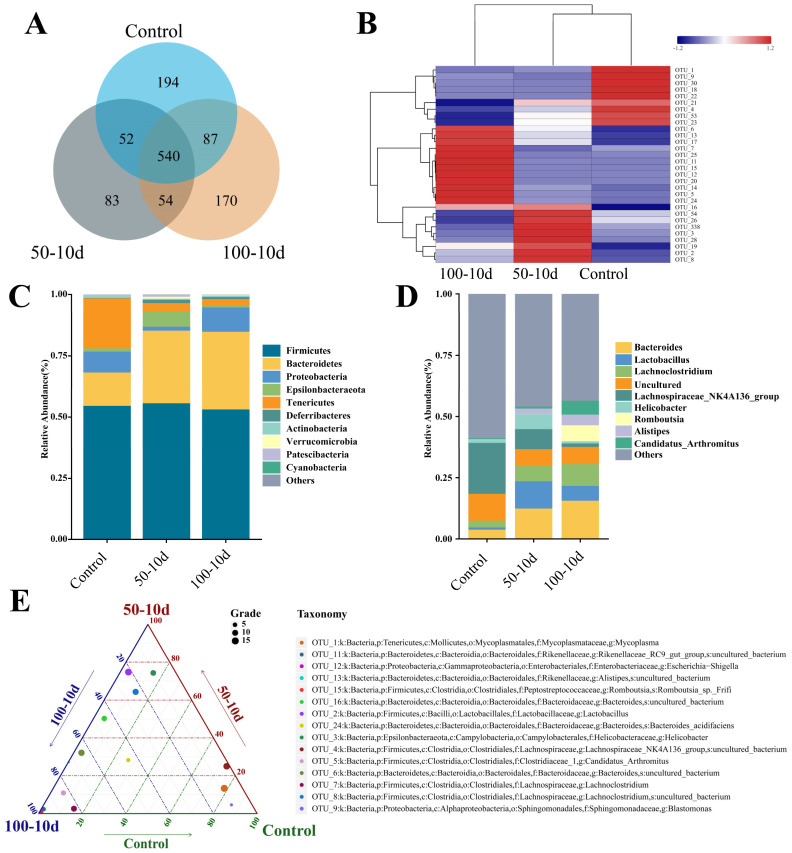
Differences in gut flora composition after treatment with 50 mg/kg and 100 mg/kg of 2-BP on 10th day. (**A**) Number of strains shared and endemic among the three groups. (**B**) Heat map of differential intestinal flora. Red color indicates a higher number of OTUs detected, while blue color indicates a lower number. (**C**) Composition of intestinal flora at the level of phylum. (**D**) Composition of intestinal flora at the level of genus. (**E**) Ternary phase diagrams revealed the species of bacteria with the most significant differences between groups. The size of the dots indicates the relative number of OTUs. The closer to the apex of the triangle indicates more significant differences.

For mice injected with 2-BP for 10 days, the Venn diagram shows the number of different OTUs (Operational Taxonomic Units) in the sequencing results of the intestinal flora of mice in each group (Figure 1A). The abundance composition of the top 10 OTU-representative sequences in each group is shown in an abundance stacked plot in Figure 1C,D. The top 15 out-representative sequences in terms of abundance are then further analyzed in a ternary phase diagram. In the 50-10d group, *Firmicutes*, *Bacteroidetes*, and *Proteobacteria* are significantly enriched. In contrast, the 100-10d group is notably enriched in *Romboutsia*, *Rikenellaceae_RC9_gut_group*, *Candidatus_Arthromitus*, and *Escherichia-Shigella*.

**Figure 2:**
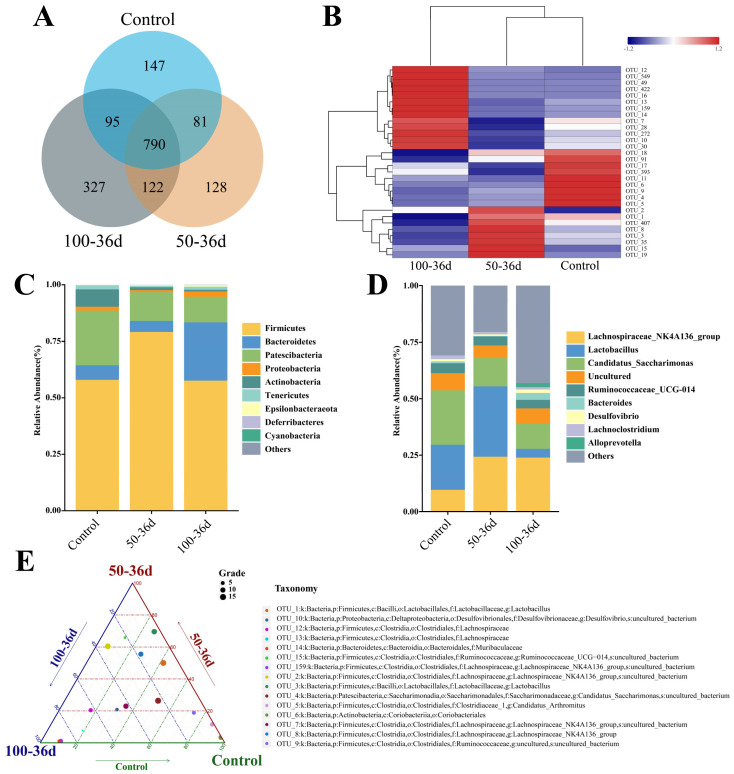
Differences in gut flora composition after treatment with 50 mg/kg and 100 mg/kg of 2-BP on 36th day. (**A**) Number of strains shared and endemic among the three groups. (**B**) Heat map of differential intestinal flora. Red color indicates a higher number of OTUs detected, while blue color indicates a lower number. (**C**) Composition of intestinal flora at the level of phylum. (**D**) Composition of intestinal flora at the level of genus. (**E**) Ternary phase diagrams revealed the species of bacteria with the most significant differences between groups. The size of the dots indicates the relative number of OTUs. The closer to the apex of the triangle indicates more significant differences.

**Figure 3:**
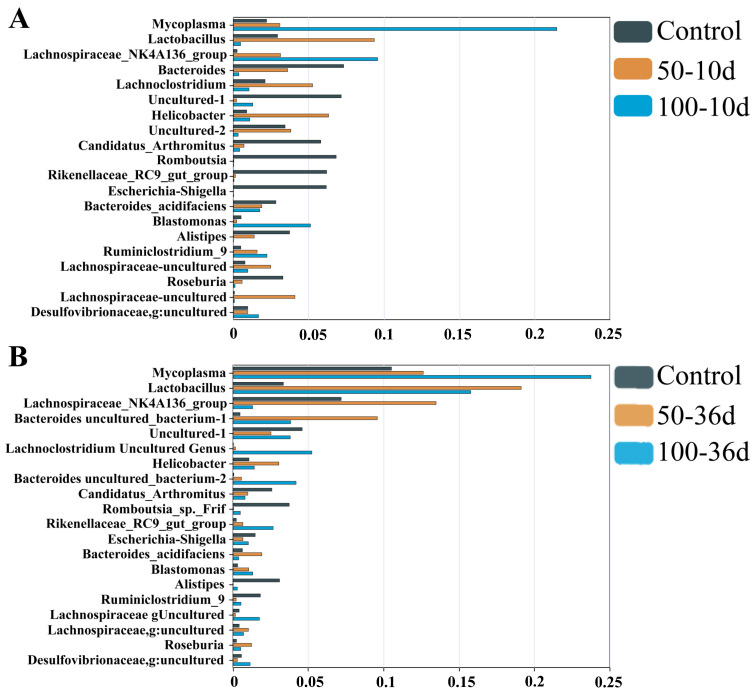
The most significant differences in bacteria following 2-BP injection. (**A**) Day 10. (**B**) Day 36. The vertical coordinate is the name of the bacteria and the horizontal coordinate represents its relative abundance.

For mice injected with 2-BP for 36 days, the Venn diagram shows the number of different OTUs in the sequencing results of the intestinal flora of mice in each group (Figure 2A). The species richness information of the diverse groups of enteric flora was then further categorized and the abundance composition of the top 10 OTU-representative sequences in each group has been depicted in the abundance stacking diagram (Figure 2C,D). Compared with the control group, it is clearly observed from the figure that the treatment with 100 mg/kg 2-BP leads to a significant reduction in the abundance of *Candidatus_Saccharimonas* and lactic acid bacteria on day 36. At the same time, the abundances of *Muribaculaceae*, *Lachnospiraceae_NK4A136_group*, *Alistipes of NK4A136_group*, and *Lachnospiraceae* are significantly increased (Figure 2E). The overall changes in intestinal microbial composition after 2-BP treatment can be seen in Figure 3.

#### 2.2.3. Prediction of Intestinal Differential Flora Function

**Figure 4:**
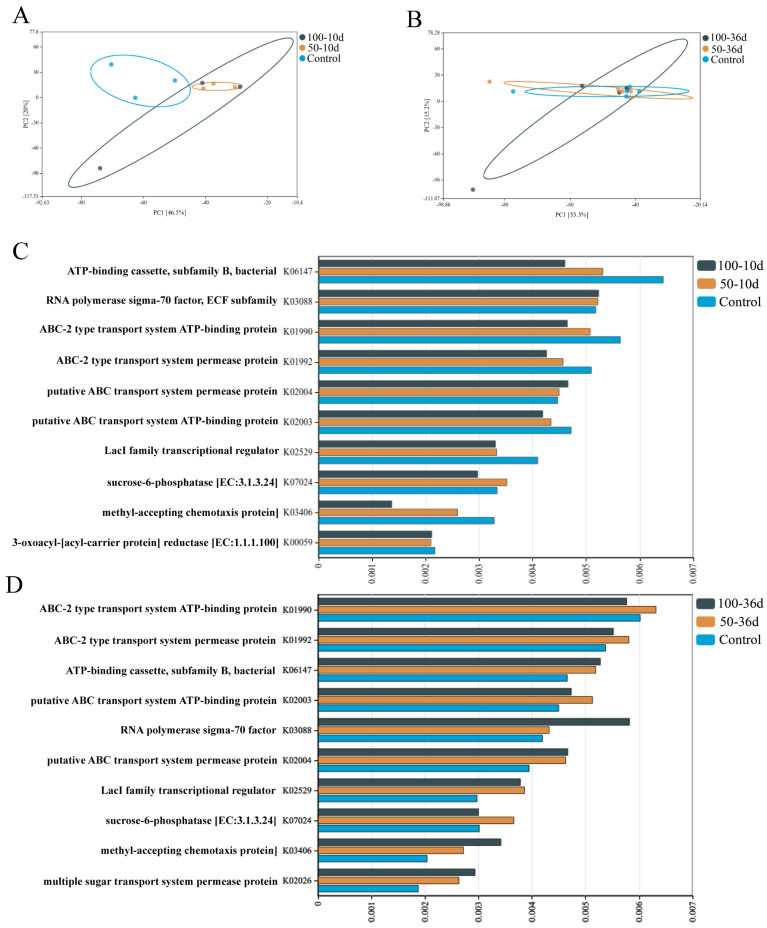
Predictions of intestinal flora function. (**A**,**B**) PC1 analysis of gut flora function on day 10 (**A**) and day 36 (**B**). The higher the sample similarity, the closer the points are. Circles represent the homogeneity of the samples; the smaller the circle, the higher the homogeneity. (**C**,**D**) Histogram of predicted intestinal flora function on day 10 (**C**) and day 36 (**D**). The vertical coordinate is the function and the horizontal coordinate is the relative number of OTUs with that function.

On the 10th day, the gut microbiota of mice in the treatment group who received 100 mg/kg of 2-BP displayed a distinct separation from the control group (Figure 4A). Additionally, Kyoto Encyclopedia of Genes and Genomes (KEGG) functional enrichment analysis revealed that 2-BP treatment caused a significant reduction in the expression of K04147 (Dopamine D4 receptor), K01990 (ABC-2 type transport system ATP-binding protein), K02003 (ABC transport system and ATP-binding protein), K02529 (LacI family transcriptional regulator), and K03406 (Methyl-accepting chemotaxis protein) (Figure 4C).

However, on day 36, there was no significant difference shown in the gut microbiota function prediction (Figure 4B). But, contrary to the function prediction of the samples on day 10, the differences in some metabolic functions of the 100-36d group compared to the control group were manifested as an increase, while in the 100-10d group on day 10, the differences manifested as a decrease, particularly for RNA generation (K03088), methylation (K03406), and polysaccharide transport pathways (Figure 4D).

### 2.3. Effects on Male Reproduction in Mice

#### 2.3.1. Visceral Coefficient and Morphology of Testis and Epididymis in Mice

It was found that on both day 10 and 36, mice in the 100 mg/kg 2-BP groups displayed a substantial accumulation of fat attached to the epididymides (Figure S7A) and the testes were enveloped in adipose tissue, making it challenging to separate them (Figure S7B).

#### 2.3.2. The Histology of Testis and Epididymis

**Figure 5:**
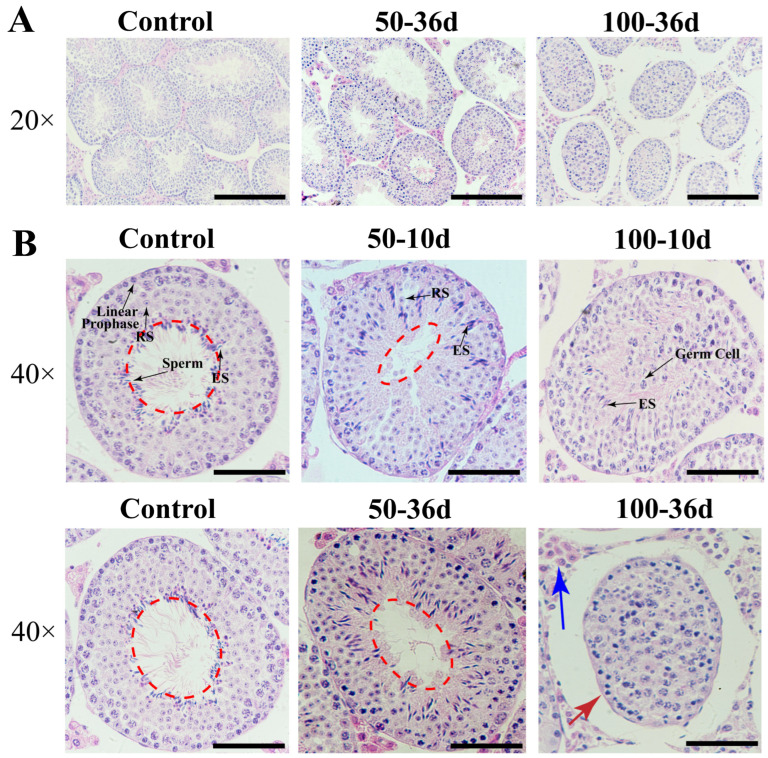
Testicular tissue HE staining on day 10 and day 36, respectively, after treatment with 50 mg/kg and 100 mg/kg of 2-BP. The circle composed of red dashed lines represents the area of seminiferous tubule. The blue arrow indicates testicular interstitial cells. The red arrow indicates seminiferous tubules. The seminiferous tubules are far away from the testicular interstitial cells. ((**A**): 20× objective lens, scale = 200 μm; (**B**): 40× objective lens, scale = 100 μm.)

In the 100-36d group, the lumens of seminiferous tubules disappeared and germ cells were arranged in a disorganized manner, with unclear staging of spermatogenesis, the absence of mature spermatozoa, and the presence of multinucleated giant cells (Figure 5). The histological structure of the epididymis in the 50-36d group was similar to the control group, indicating no significant differences, but the 100 mg/kg 2-BP treatment group exhibited a decreased density and quantity of the stored spermatozoa (Figure S8).

**Figure 6:**
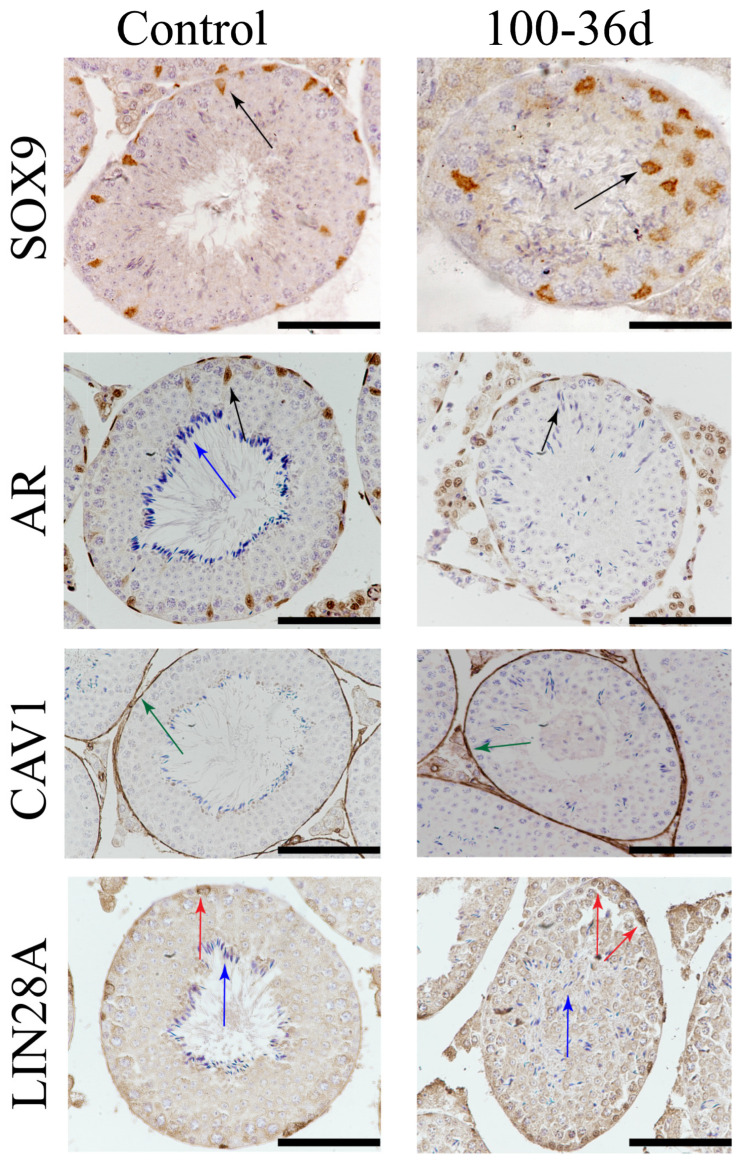
Testicular tissue IHC staining on day 36 after treatment with 100 mg/kg of 2-BP. For SOX9: in the 100-36d group, the Sertoli cells are arranged in disorder and are away from the basal membrane side of the seminiferous tubules and migrate to other positions in the lumen (the black arrow indicates the Sertoli cells in abnormal positions). For AR: the expression signal of germ cells in the lumen is weak (black arrow), the germ cells are arranged irregularly (blue arrow), and the sperm cells are not arranged around the regular lumen. For CAV1: the difference between the two groups (green arrow) is not significant, but the germ cells in the seminiferous tubules of the 100-36d group are arranged in disorder. For LIN28A: the difference between the two groups is not significant (red arrow), but the germ cells in the seminiferous tubules of the 100-36d group are arranged in disorder (blue arrow). (40× objective lens; scale = 100 μm).

Interestingly, in the control group, the supporting cells labeled with SOX9 were found to be uniformly distributed along the basement membrane of the seminiferous tubules. However, in the 100 mg/kg 2-BP treatment group, local aggregations of SOX9-labeled supporting cells were observed that had migrated away from the germinal epithelium and showed enhanced SOX9 expression. The supporting cells, peritubular myoid cells, and testicular interstitial cells in the control group expressed androgen receptors (ARs). However, in the group treated with 2-BP at a dose of 100 mg/kg, there was a potential decrease in AR expression, specifically in the supporting cells. Cav-1 expression was observed in both the testicular interstitium and peritubular epithelial cells, with no significant differences found between the 2-BP treatment groups and the control group (Figure 6).

#### 2.3.3. Sperm Morphology and Motility

**Figure 7:**
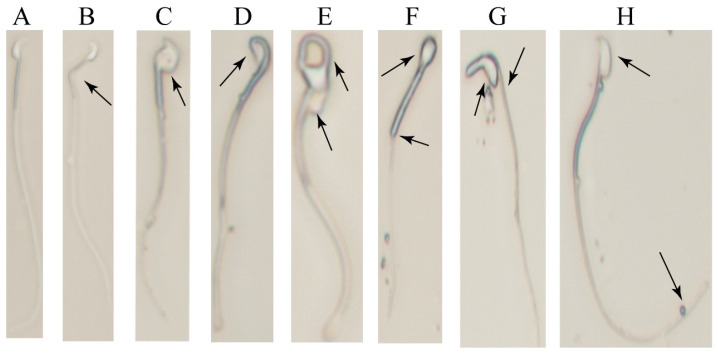
Typical diagram of aberrant spermatozoa appearing after 100 mg/kg 2-BP injection. (**A**) Normal sperm morphology; (**B**) Sperm neck bending; (**C**) Abnormally enlarged sperm head; (**D**) Sperm neck bending and abnormal head morphology; (**E**) Incomplete shedding of sperm cytoplasm; (**F**) Abnormal sperm head; (**G**) Irregular sperm head; (**H**) Abnormally curled sperm tail.

Figure 7A illustrates the normal sperm morphology with increased sperm deformities and appearance of the neck curvature (Figure 7B,D) in spermatozoa of the 2-BP group, as well as the large head (Figure 7C), cytoplasm (not completely detached) (Figure 7E), round head (Figure 7F), irregular head (Figure 7G), and curly tail (Figure 7H) types.

**Figure 8:**
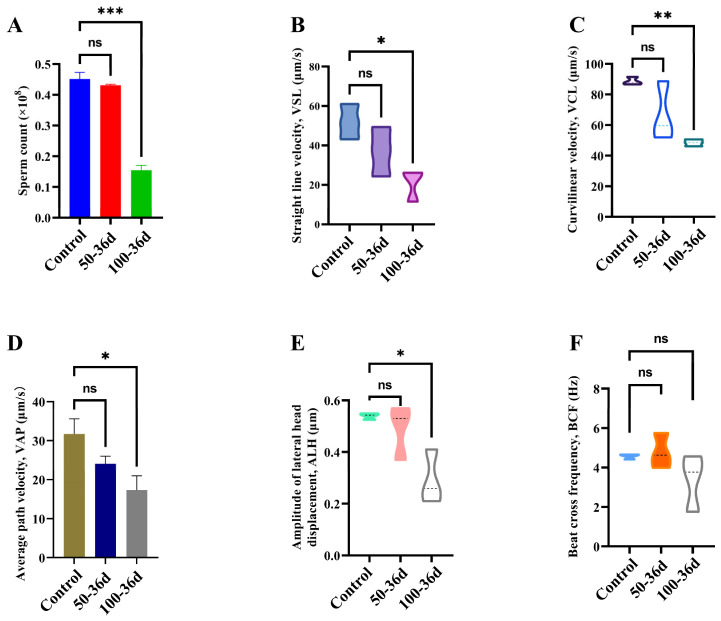
Sperm viability was assayed on day 36 after 100 mg/kg 2-BP injection. (**A**) Sperm count; (**B**) Straight-line velocity; (**C**) Curvilinear velocity; (**D**) Average path velocity; (**E**) Lateral amplitude; (**F**) Beat frequency. Significance levels were denoted as * for *p* < 0.05, ** for *p* < 0.01, *** for *p* < 0.001 and non-significant (ns).

It was observed that on 10th day, there were no significant differences found in the sperm count and motility between the 2-BP injection group and the control group (Figure S9). However, on the 36th day, the 100-36d group exhibited a significant decrease in the sperm count, straight-line velocity (VSL), curvilinear velocity (VCL), average path velocity (VAP), and amplitude of lateral head displacement (ALH) in comparison to the control group (Figure 8).

#### 2.3.4. Expression of mRNA in Testis

**Figure 9:**
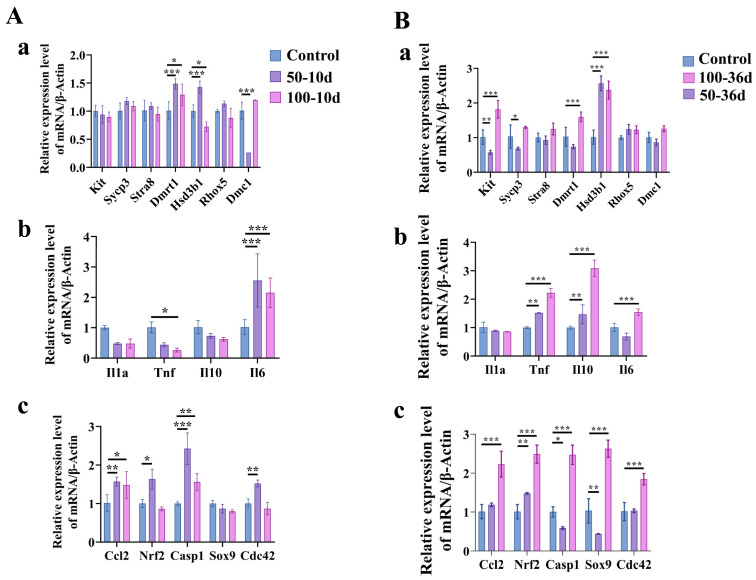
Changes in testicular gene expression in mice after 2-BP injection. (**A**) Changes in gene expression on day 10; (**a**) Expression levels of genes related to spermatogenesis; (**b**) Expression levels of inflammatory factors; (**c**) Expression levels of some chemokine genes. (**B**) Changes in gene expression on day 36. (**a**) Expression levels of genes related to spermatogenesis; (**b**) Expression levels of inflammatory factors; (**c**) Expression levels of some chemokine genes. Significance levels were denoted as * for *p* < 0.05, ** for *p* < 0.01, *** for *p* < 0.001.

The expression of *Dmrt1*, *Ccl2*, and *Casp1* mRNA in the testes was significantly upregulated in the 2-BP treatment groups (*p* < 0.05). The 50-10d group demonstrated significant upregulation of *Hsd3b1*, *Nrf2*, and *Cdc42* mRNA expression (*p* < 0.01), while expression of Dmc1 was significantly downregulated (*p* < 0.0001). On the contrary, the 100-10d group exhibited significant downregulation of *Hsd3b1* mRNA expression (*p* < 0.05). Additionally, expression of *IL-6* mRNA was upregulated in all 2-BP treatment groups on day 10 (*p* < 0.001), but that of *TNF-α* was downregulated in the 100 mg/kg 2-BP treatment group (*p* < 0.05) (Figure 9A).

On day 36, it was observed that in comparison to the control group, the testes of the 2-BP treatment groups displayed significant upregulation of *Hsd3b1* mRNA expression. The 50-36d group showed significant downregulation of *Sox9*, *Casp1*, *Kit*, and *Sycp3* mRNA expression but significant upregulation of *Nrf2* mRNA expression. However, the 100-36d group demonstrated significant upregulation of *Ccl2*, *Nrf2*, *Casp1*, *Sox9*, *Cdc42*, *Kit*, and *Dmrt1* mRNA expression. In addition, on day 36 after injection, expression of both *TNF-α* and *IL-10* was upregulated in the 2-BP treatment groups, whereas that of *IL-6* was upregulated in the 100-36d group and downregulated in the 50-36d group (Figure 9B).

## 3. Discussion

Although 2-BP has been used as an inhibitor to explore the role of palmitoylation in spermatogenesis and maintenance of sperm function, in the present study, the exploration of the reproductive impairment associated with 2-BP injection remains innovative. The findings of this study indicate that 2-BP caused disruption in the reproductive system in male mice. Moreover, both the IF and IHC results indicated that β-catenin and CAV1 in the control group and 2-BP group remained intact, thus indicating that the germinal epithelial barrier was not damaged. The results of qRT-PCR indicated that in the 100-36d group, the expression of *Dmrt1* mRNA showed a significant increase, while the expression of *Dmc1* and *Sycp3* was markedly decreased, suggesting that the spermatogonia entering the process meiosis were reduced and that 2-BP could exert a pronounced inhibitory effect on meiosis, thus affecting both sperm quantity and quality [42,43]. Combining the results of IHC and sperm motility detection, the toxicity of 2-BP on the male reproductive system of mice needs a certain period of time to manifest, and the impact in a short time is relatively small. However, this study only focused on the phenomenon of its toxicity, and the mechanism of how it produces a toxic effect by inhibiting palmitoylation still needs further exploration.

Furthermore, the toxicity of 2-BP not only impacts the reproductive system but also leads to an elevation in the expression levels of inflammatory factors in the kidney and spleen, whereas the inflammatory response in the liver is relatively mild. We analyze that the intestinal microbiota play a certain role. The enrichment of *Muribaculaceae* and *Alistipes* in the intestines of the 100 mg/kg group mice on the 36th day could be potentially associated with the inhibitory effect of 2-BP on liver inflammation. There are studies indicating that *Muribaculaceae* can effectively reduce colonic barrier dysfunction to improve liver injury [44]. *Alistipes*, a member of the *Bacteroidetes* phylum, has demonstrated beneficial effects in conditions such as inflammatory bowel disease, inflammatory liver cirrhosis, and inflammatory NASH/NAFLD by producing propionate and acetate [45]. It was observed that on the 36th day, liver cells exhibited distinct features of fatty degeneration and hepatocyte swelling, possibly due to the inhibitory effect of 2-bromo palmitic acid ester on lipid breakdown. Changes in the intestinal flora, therefore, protect the internal organs and minimize the effects of 2-BP. This regulation may be systemic, in contrast to previous findings that inhibition of palmitoylation did not cause a large-scale inflammatory response [46].

The alterations in the gut microbiota are often attributed to factors such as diet. However, the changes in the gut microbiota induced by intraperitoneal injection of 2-BP, as observed in this study, represent a relatively novel finding. A study has shown that 2-BP can lead to the development of intestinal adhesions and stenosis, thereby restricting the mobility of intestinal contents and promoting bacterial overgrowth [47]. The gut microbiota of mice treated with 100 mg/kg 2-BP on day 10 demonstrated an enrichment of bacteria belonging to the phylum *Firmicutes*, the order *Clostridiales*, and the family *Rikenellaceae* based on the results of 16s rDNA sequencing. In the human gut, these microbial taxa have been identified to contain the chaperone B subunit of the mitochondrial matrix protease Clp (*CLPB*) gene, which is negatively associated with body mass index, waist circumference, and total fat mass [48].

However, bacteria harmful to the animals were detected to be enriched after injection of 2-BP. Most members of the genera *Bacteroides* and *Desulfovibrio* are capable of producing LPS. *Desulfovibrio* spp., a genus of sulfate-reducing bacteria, was found to be enriched in the intestines of mice treated with 50 mg/kg 2-BP on the 36th day. This genus could potentially play a significant role in the progression of liver steatosis triggered by 2-BP. Interestingly, in mice subjected to intraperitoneal injection of 2-BP, the relative abundance of *Escherichia-Shigella*, *Bacteroides* and their subgroups in the intestine was found to be significantly increased, leading to splenomegaly, organ inflammation, and injury [49]. The substantial increase in *Proteobacteria* and species of the family *Desulfovibrionaceae* caused by 2-BP treatment can lead to gut dysbiosis in chronic kidney disease [50].

Interestingly, in the present study, 2-BP injection caused mycoplasma to proliferate in the intestinal flora. Mycoplasma, a bacterial virus that triggers an inflammatory response in animals [51], is often studied as a pathogen and is closely related to the intestinal flora [52,53]. However, there is still a gap in the research related to the effect of mycoplasma by palmitoylation, which will be part of our future research direction. In summary, 2-BP injection resulted in dysfunction of the male reproductive system and reduced sperm viability in mice. The inflammatory response was enhanced in the spleen and kidney, while changes in the intestinal flora played a role in alleviating liver damage. Although this study indicated that intraperitoneal injection of 2-BP had extensive systemic impacts on mice, the mechanisms of its action and the functions of the gut microbiome still require further exploration for validation, which is also one of our future research directions.

## 4. Materials and Methods

### 4.1. Experimental Animals and Reagents

Healthy 8-week-old male ICR mice were procured from the animal center of the Fourth Military Medical University. They were then placed in a controlled environment (temperature ranging between 22 °C and 24 °C, a 12 h light/12 h dark cycle, with free access to food and water). The body weight of each mouse was recorded before injection and they were administered intraperitoneal injections of 2-BP (CAS. No. 18263-25-7, Sigma-Aldrich, Steinheim, Germany) at doses of 0, 50, and 100 mg/kg (drug mass/mouse body weight), once per day for 5 days (the preparation method of 2-BP solution can be seen in the Supplementary Materials). The control group was injected with the solvent once a day for five days. Each group consisted of 8 mice, with day 0 being designated as the first day for 2-BP injection. The mice were executed and test samples were collected on days 10 and 36, respectively. The samples were labeled as 50-10d and 100-10d on the 10th day and 50-36d and 100-36d on the 36th day.

### 4.2. Tissue RNA Extraction and cDNA Reverse Transcription

Total RNA was extracted from each tissue using TRIzol reagent (Invitrogen, Carlsbad, CA, USA). The concentration and purity of RNA were analyzed with a Nanodrop 2000 spectrophotometer (Thermo Fisher Scientific, Shanghai, China) (with an OD260/OD280 ratio > 1.8). Subsequently, 1 μg of high-quality RNA was reverse transcribed into cDNA using a complementary DNA (cDNA) synthesis kit (Code RR014A, TaKaRa, Osaka, Japan) for qRT-PCR. The entire process of reverse transcription was performed using a reverse transcription kit (Code No. 611139ES60, YEASEN, Shanghai, China) according to the manufacturer’s instructions.

### 4.3. Quantitative Real-Time Polymerase Chain Reaction

The gene-specific primers were synthesized by Sangon Biotech Co., Ltd. (Shanghai, China). The qRT-PCR program was carried out using the 2×SYBR Green qPCR Master Mix reagent and BIO-RAD iQ™5 quantitative PCR system (BIO-RAD Inc., Hercules, CA, USA). Each experiment was performed with triplicate replicates. The quantitative RT-qPCR program comprised the following steps: 95 °C for 5 min, 95 °C for 5 s, 60 °C for 5 s, and 72 °C for 25 s. This process was repeated for 40 cycles. The 2^−ΔΔCt^ method was used to determine the relative mRNA expression levels of each gene, with the control group as the reference and *β-actin* as the housekeeping gene. The sequences of specific primers used for the quantification of inflammation-related genes such as *IL-1a*, *TNF-a*, *IL-10*, *IL-6*, and *β-actin* are depicted in Table 1.

### 4.4. Collection and Preservation of Intestinal Contents

Prior to the sampling process, mice were subjected to a 12 h fasting period. After anesthesia, intestine segments were carefully isolated. These segments were then gently rinsed with pre-chilled physiological saline and excess moisture was removed using absorbent paper. Intestinal contents from the ileum and cecum were then collected by gently scraping the intestinal mucosa with glass slides. The collected contents were mixed and transferred into a sterile tube. After rapid freezing in liquid nitrogen, the samples were subsequently stored at −80 °C. The frozen contents were stored and mailed in dry ice. The 16s rDNA sequencing analysis was carried out by Novogene Co., Ltd. (Shanghai, China). The specific steps of analysis can be found in the Supplementary Materials.

### 4.5. Hematoxylin and Eosin (HE) Staining and Immunohistochemistry (IHC)

The liver, kidneys, and one side of the testis and epididymis of mice were fixed with 4% paraformaldehyde-treated tissue and then dehydrated and cleared in xylene until they became transparent (the specific steps can be found in the Supplementary Materials). Subsequently, the tissues were embedded in paraffin and all the paraffin blocks were sectioned into slices of 5 μm thickness and mounted on slides. A portion of these sections was designated for HE staining, while the remainder was reserved for IHC. For HE staining, the protocol encompassed deparaffinization, rehydration, and staining with hematoxylin and eosin. Thereafter, the tissue morphology was observed under a microscope (Nikon Ni-U, Tokyo, Japan) post sealing. IHC was employed to determine the localization of Sox9 (ab185966, Abcam, Cambridge, UK), AR (ab273500, Abcam), Cav-1 (ab32577, Abcam), and LIN28A (ab279647, Abcam) in the testicular tissues to analyze the testicles for pathological changes. The initial steps of the IHC experimental procedure involved deparaffinization, rehydration, and permeation (these steps are the same as those in HE staining). We used a citrate antigen retrieval solution (50×, C1032-100 mL, Solarbio, Beijing, China) and repaired the samples in a water bath at 98 °C for 25 min. Subsequently, the tissues were incubated in 3% hydrogen peroxide (H_2_O_2_) solution for 10 min to inhibit the endogenous peroxidase activity. The tissues were then blocked with 5% bovine serum albumin (BSA) for 2 h and washed with PBS three times (5 min per wash). The primary antibodies against Sox9, Cav-1, AR, and LIN28A (10 ng/μL) were diluted in Dako antibody diluent. The tissue sections were incubated with the above-indicated primary antibodies overnight at 4 °C. After washing, secondary antibody (ab288151, Abcam) labeling was conducted by incubating the tissues with the secondary antibody for 1 h at 25 °C. Finally, post 3,3′-diaminobenzidine (DAB) staining, the tissue sections were kept in the dark for 5 min and counterstained with hematoxylin reagent for 3 min.

### 4.6. Sperm Motility

The computer-aided sperm analysis system (CASA; HVIEW, Shenzhen, China) is a widely used system for analyzing sperm motility indices and was used to analyze sperm motility. Sperm (1 m/L) harvested from the epididymal cauda was placed in the sperm dilution reagent and incubated at 37 °C for 5 min before assessing motility [54].

### 4.7. Statistical Analysis

GraphPad Prism software (Version 8.0) was employed for the statistical analyses. The data have been presented as mean ± standard deviation (SD), and an unpaired *t*-test was used for the statistical comparisons. Significance levels were denoted as * for *p* < 0.05, ** for *p* < 0.01, *** for *p* < 0.001, **** for *p* < 0.0001 and non-significant (ns). The analysis methods related to gut microbiota can be found in the Supplementary Materials.

### 4.8. Animals Ethics

The handling of mice was conducted in accordance with the Animal Care and Use Committee of Northwest Agriculture and Forestry University (permit number: 17-347, data: 2017-10-13) and the Government of China Principles for the Utilization and Care of Vertebrate Animals Used in Testing, Research, and Training.

## 5. Conclusions

In summary, this study examined the potential effects of intraperitoneal injection of 2-BP at doses of 50 mg/kg and 100 mg/kg on viscera and spermatogenesis in 8-week-old male mice. It was observed that intraperitoneal injection of both 50 mg/kg and 100 mg/kg 2-BP promoted inflammation in the spleen and kidneys of mice. 2-BP induced changes in the gut microbiota of mice, which could be directly correlated with decreased body weight, improved or exacerbated liver inflammation, hepatic steatosis, and testicular inflammation, as well as extensive spleen and kidney damage. A dose of 100 mg/kg 2-BP exhibited reproductive toxicity in male mice, leading to abnormal testes and disrupted arrangement of the germ cells. Additionally, 2-BP was able to inhibit the process of meiosis, leading to abnormal sperm development, a reduction in sperm motility, and an increase in sperm deformities.

## Figures and Tables

**Table 1:** Quantitative primers for RT-qPCR.

Gene Name	Primer Sequence (5′-3′)	Product Length (bp)
*IL1-β*	F: GAGCCGGGTGACAGTATCAG R: GCTGATCTGGGTTGGATGGT	141
*Tnf-α*	F: AGCCGATGGGTTGTACCTTG R: ATAGCAAATCGGCTGACGGT	99
*IL10*	F: TGAGGCGCTGTCATCGATTT R: TGGCCTTGTAGACACCTTGG	105
*IL6*	F: CACTTCACAAGTCGGAGGCT R: CTGCAAGTGCATCATCGTTGT	113
*Kit*	F: GCCTGACGTGCATTGATCC R: AGTGGCCTCGGCTTTTTCC	110
*Sycp3*	F: AGCCAGTAACCAGAAAATTGAGC R: CCACTGCTGCAACACATTCATA	106
*Stra8*	F: TTTGACGTGGCAAGTTTCCTG R: TAACACAGCCAAGGCTTTTGA	151
*Dmrt1*	F: CAGAGGGACGCATGGTCATC R: TGTAGTAGGCGGGGTCTGATA	96
*Hsd3b*	F: GTTGACCATGGCTGGATGGA R: CCTCCTTGGTTTCTGGTCGG	143
*Rhox5*	F: CGCAAGGTCACCAGGCTAC R: CCCCATCACCCATAGGACCA	223
*Dmc1*	F: TGCCGCTCTCCTTTCAACAT R: TCCCATGCTTCTGCAACAGG	112
*Sox9*	F: AGTACCCGCATCTGCACAAC R: ACGAAGGGTCTCTTCTCGCT	88
*Cdc42*	F: CCCATCGGAATATGTACCAACTG R: CCAAGAGTGTATGGCTCTCCAC	78
*Nrf2*	F: CCCATCGGAATATGTACCAACTG R: CCAAGAGTGTATGGCTCTCCAC	153
*Casp1*	F: ACAAGGCACGGGACCTATG R: TCCCAGTCAGTCCTGGAAATG	237
*Ccl2*	F: TTAAAAACCTGGATCGGAACCAA R: GCATTAGCTTCAGATTTACGGGT	112
*β-Actin*	F: TCTTTGCAGCTCCTTCGTTG R: TTCTCCATGTCGTCCCAGTTG	298

## Data Availability

The original contributions presented in the study are included in the article and Supplementary Materials; further inquiries can be directed to the corresponding author.

## Supplementary Materials

The following supporting information can be downloaded at: https://www.mdpi.com/article/10.3390/ijms252111415/s1.
