# Supplementary material for: 2-Bromopalmitate-Induced Intestinal Flora Changes and Testicular Dysfunction in Mice
Source: Int J Mol Sci. 2024 Oct 24;25(21):11415. doi: 10.3390/ijms252111415 (PMC11547043; doi:10.3390/ijms252111415)
Supplement: Supplementary file 1 [file ijms-25-11415-s001.zip › ijms-3230581-supplementary.pdf]

2-Bromopalmitate-Induced Intestinal Flora Changes and Testicular Dysfunction in Mice

Yuxuan Ma, Yining Chen, Long Li, Zifang Wu, Heran Cao, Chao Zhu, Qimin Liu, Yang Wang, Shaoxian Chen, Yuyan Liu and Wuzi Dong *

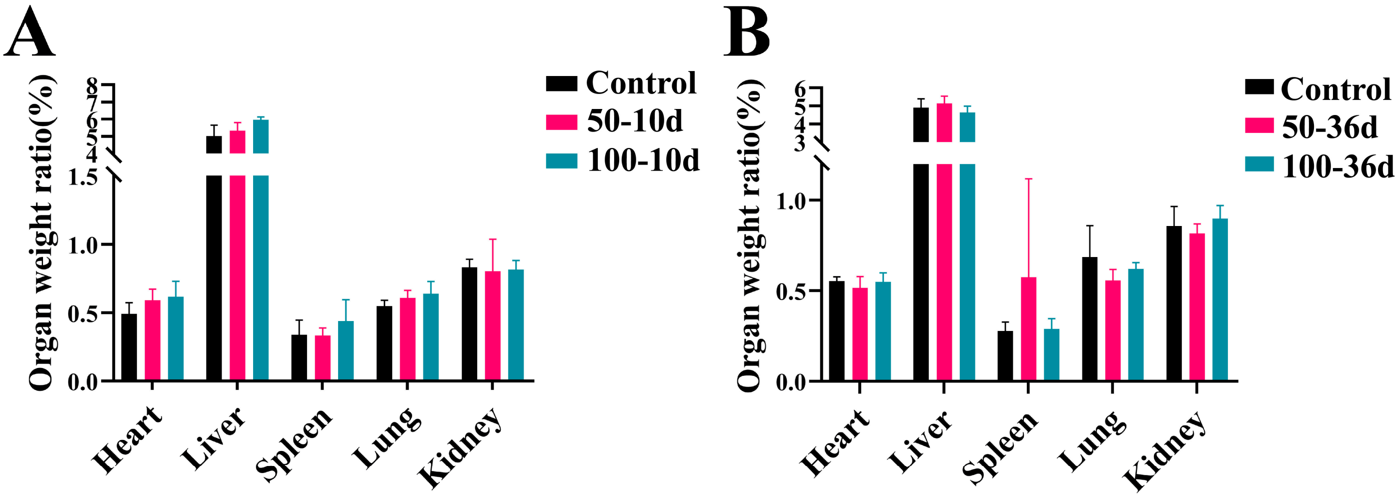

Figure S1. Percentage of organ weight 10 (A) and 36 (B) days after 2-BP injection.

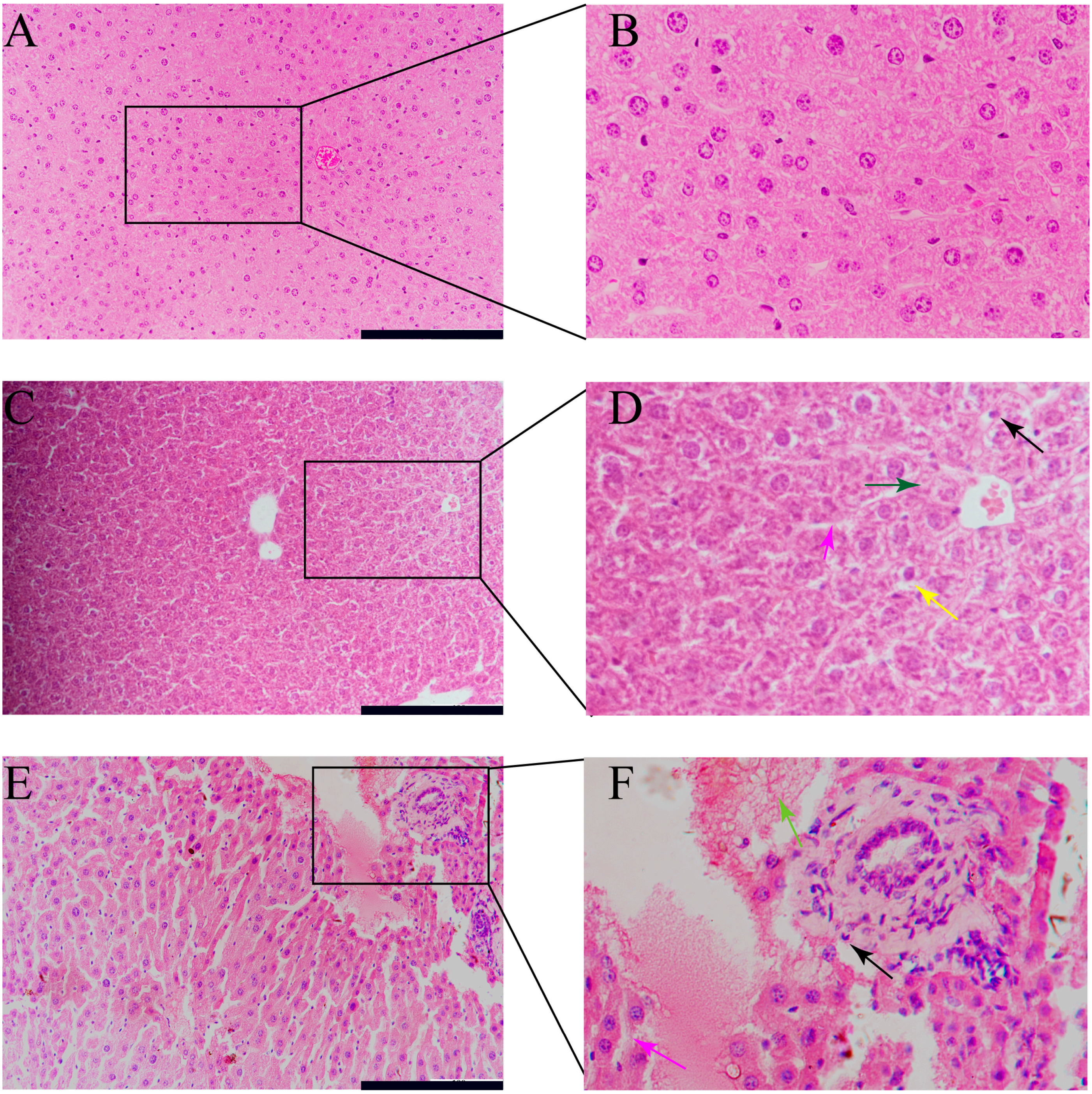

Figure S2. HE staining of mouse liver at 36 days after 100mg/kg2-BP injection. (A-B) Control group. The hepatocytes are arranged neatly, with the nuclei in the center. The nuclear membranes are sharp, and the cytoplasm is red. (C-D) 50-36d group. The hepatocytes are swollen and the liver cords are disordered. (At the place indicated by the black arrow, the hepatocytes are severely shrunken; at the place indicated by the green arrow, the hepatocytes have no normal nucleus; at the place indicated by the pink arrow, the gaps between liver cords are increased and the arrangement is disordered; at the place indicated by the yellow arrow, the hepatocytes are in the process of pathological changes (developing towards severe shrinkage). (E-F) Severe liver injury of 100mg/kg group. At the place indicated by the green arrow, the liver cords are severely damaged; at the place indicated by the pink arrow, the liver cords are incomplete; at the place indicated by the black arrow, there are white blood cells. (10X objective lens; scale=200 μm).

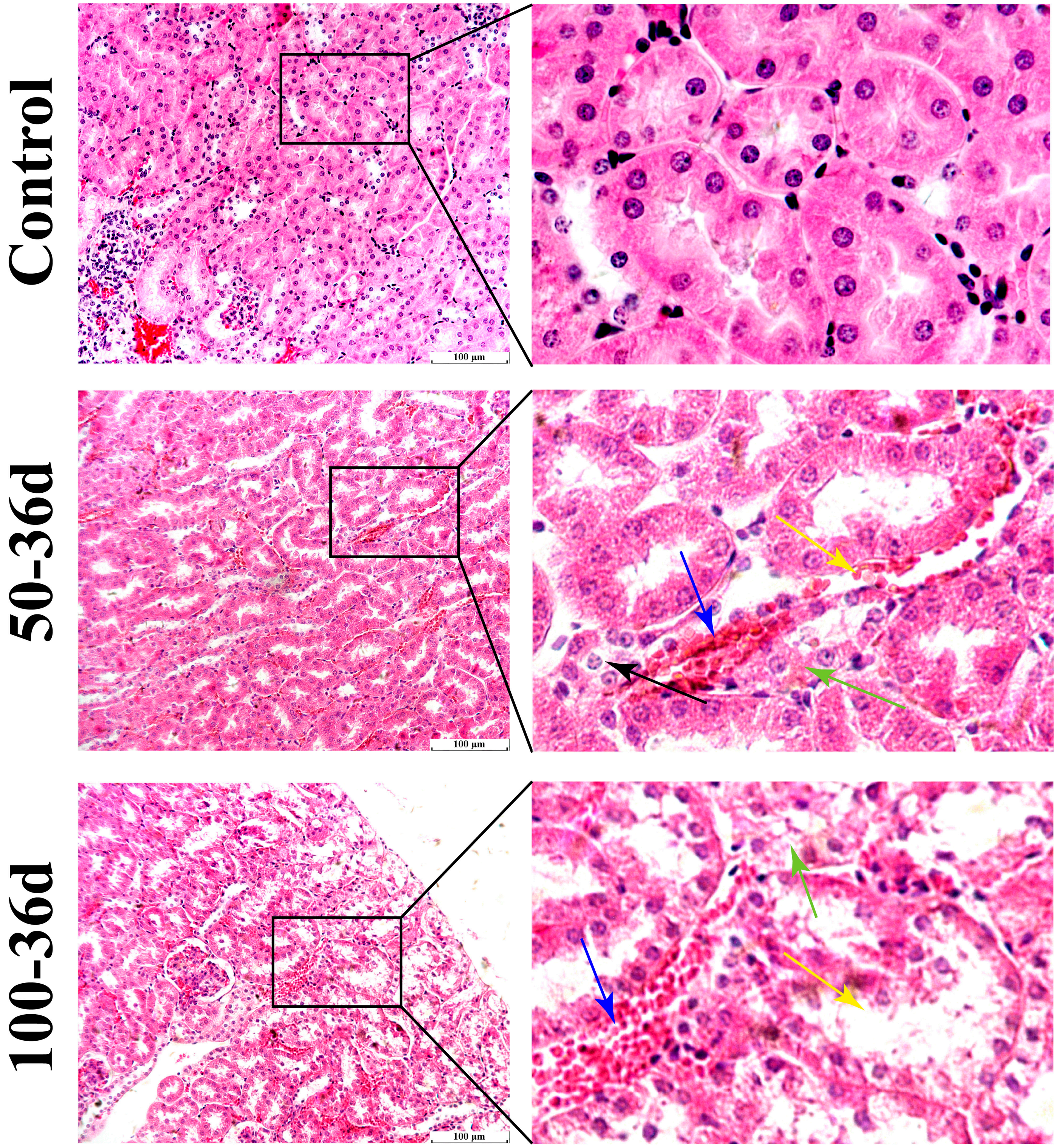

Figure S3. HE staining of mouse kidney at 36 days after 100mg/kg2-BP injection. In the Control group, the renal tubules are arranged neatly. In the 50-36d group, the kidney is damaged (black arrow: white blood cells; blue arrow: infiltration of red blood cells; yellow arrow: a small amount of red blood cells; green arrow: damaged renal tubules). In the 100-36d group, the kidney is severely damaged (blue arrow: infiltration of red blood cells; green and yellow arrows: renal tubules with different degrees of damage).

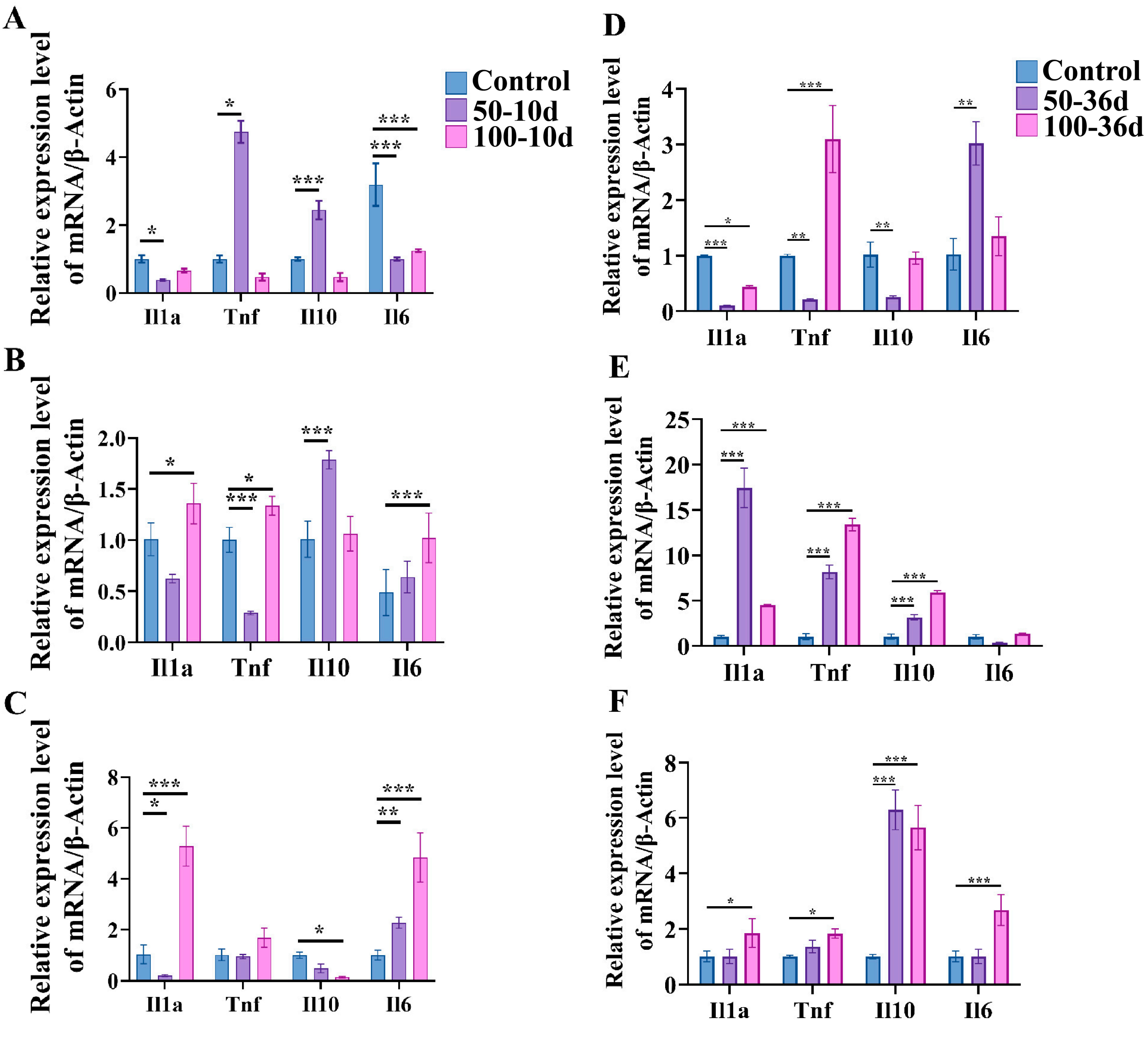

Figure S4. (A-C) Inflammatory factor expression in liver (A), spleen (B), and kidney (C) at 10 days after 2-BP injection. (D-E) Inflammatory factor expression in liver (D), spleen (E), and kidney (F) at 36 days after 2-BP injection.

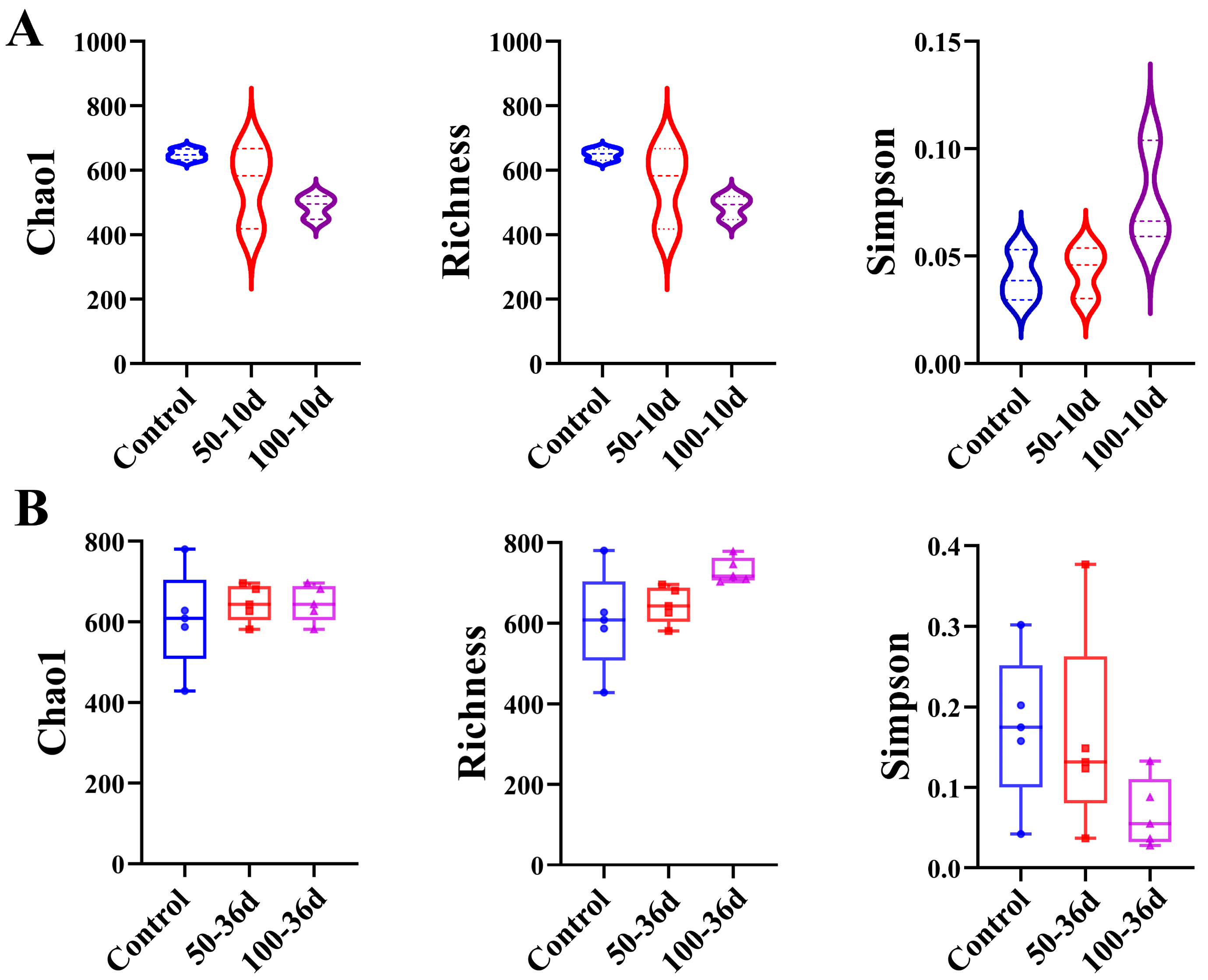

Figure S5. Changes in alpha diversity of intestinal flora in mice after 2-BP injection. (A) At 10 days after 2-BP injection. (B) At 36 days after 2-BP injection.

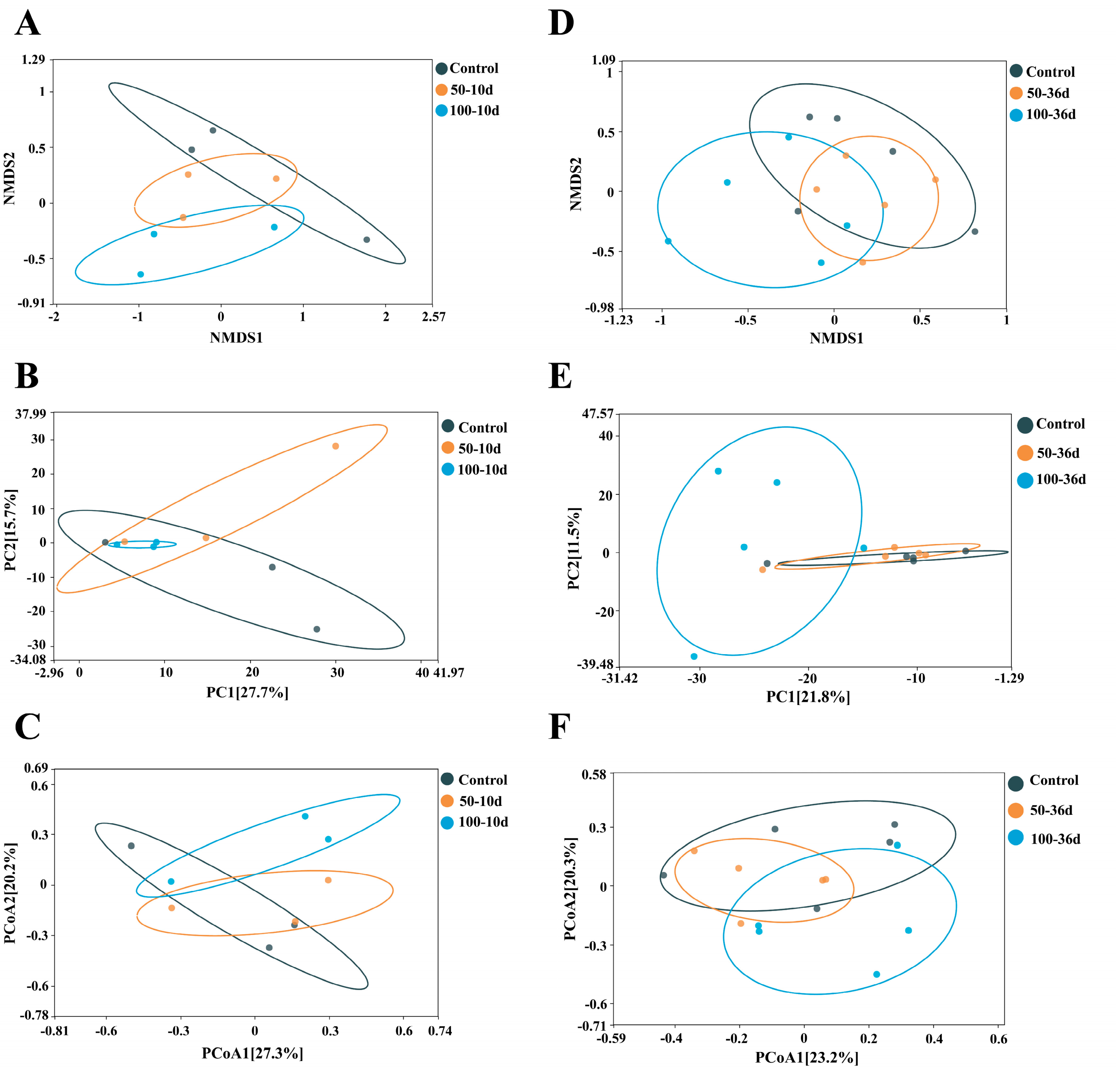

Figure S6. Changes in beta diversity of intestinal flora in mice after 2-BP injection. Each point represents a sample, with closer distances representing more similar composition. The size of the circle represents the homogeneity of the sample, the smaller the circle the more homogeneous the sample is. (A-C) The beta diversity of intestinal flora at 10 day after injection of 2-BP. (D-F) The beta diversity of intestinal flora at 36 day after injection of 2-BP.

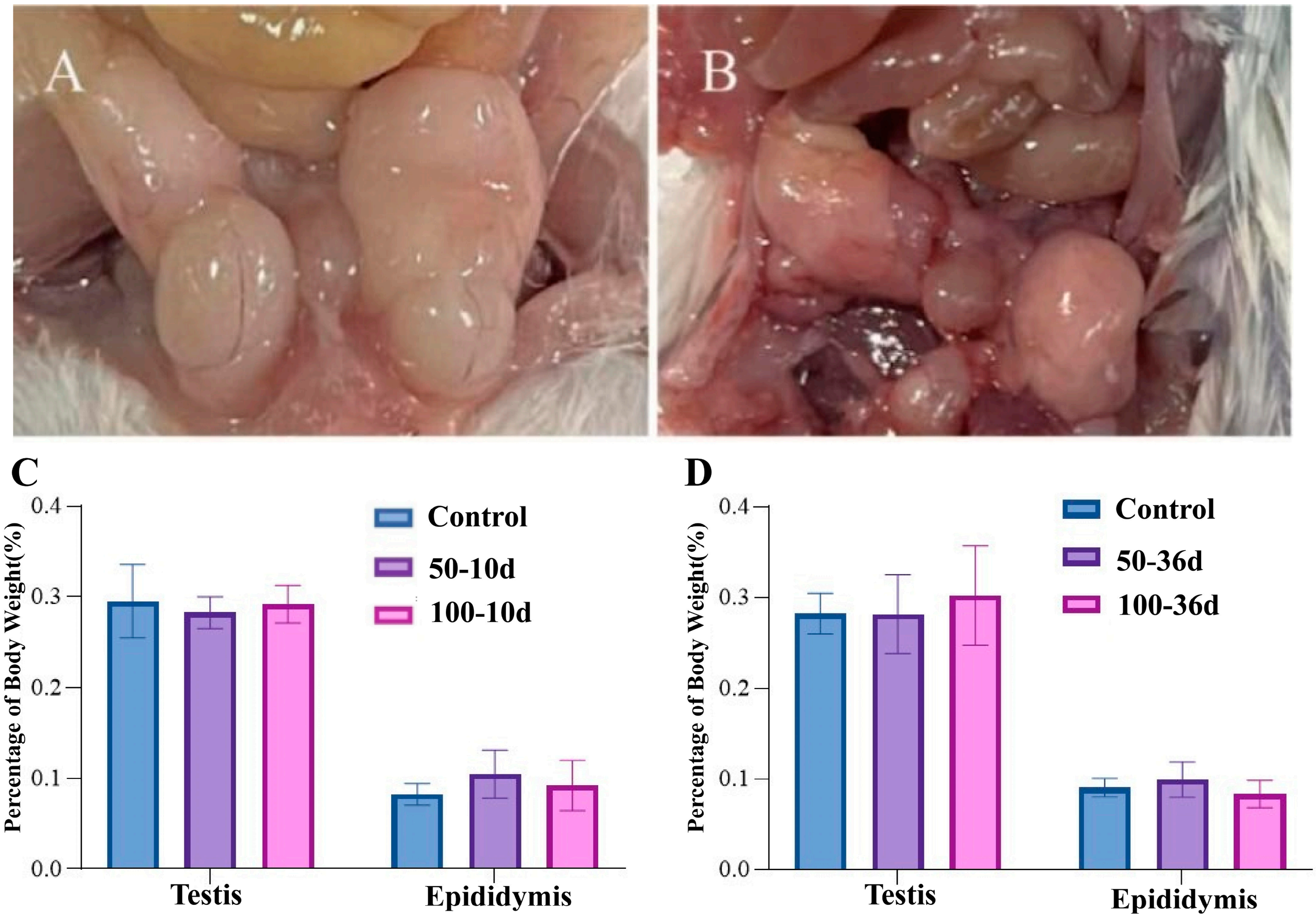

Figure S7. (A-B) Typical image of a testicle of the mouse from Control group (A) and 100-36d group (B). (C) Specific gravity of testis and epididymis in total body weight at 10 day after injection of 2-BP. (D) Specific gravity of testis and epididymis in total body weight at 36 day after injection of 2-BP.

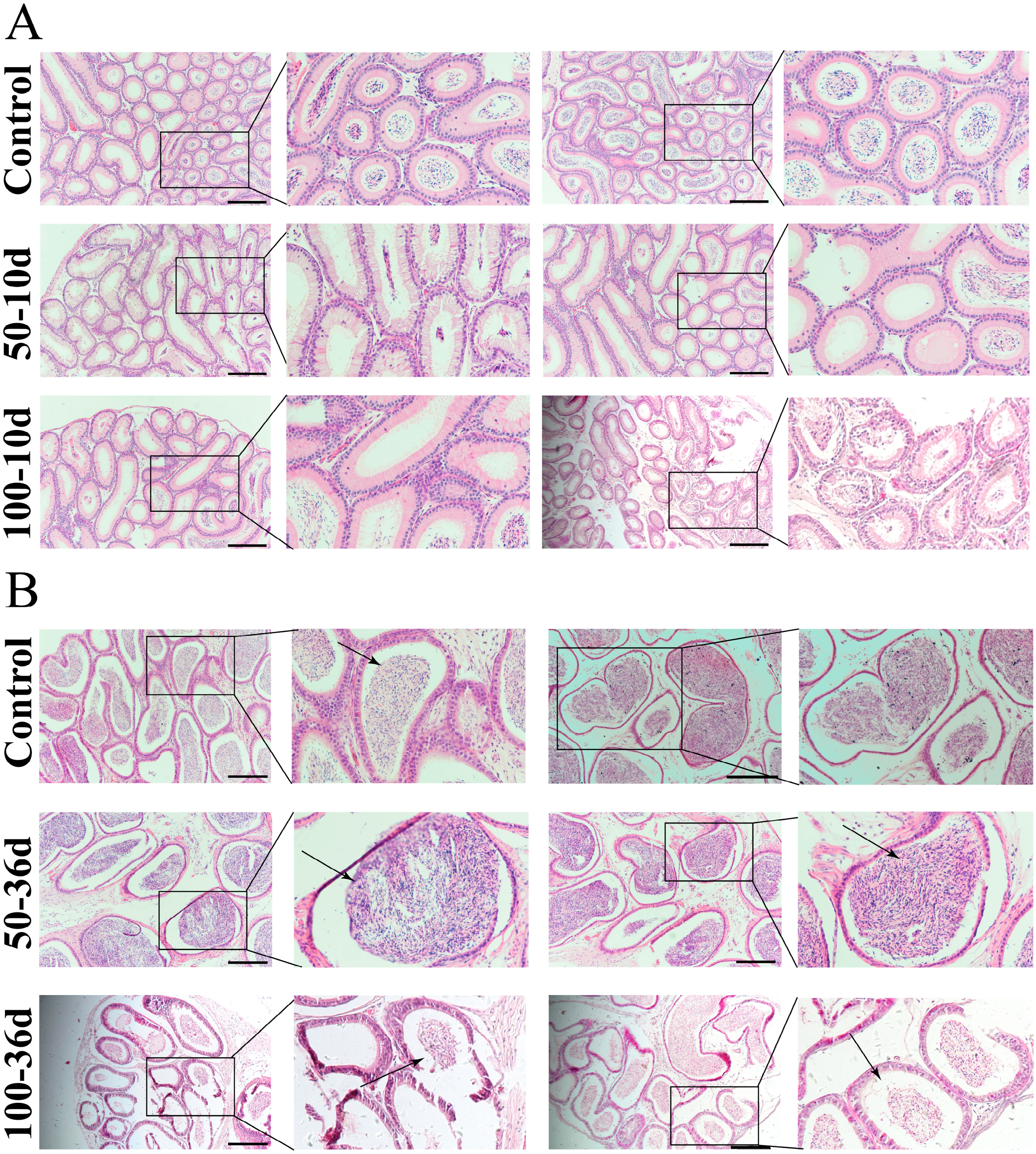

Figure S8. (A) HE staining of caput. (B) HE staining of cauda. (4X objective lens; scale=500 μm).

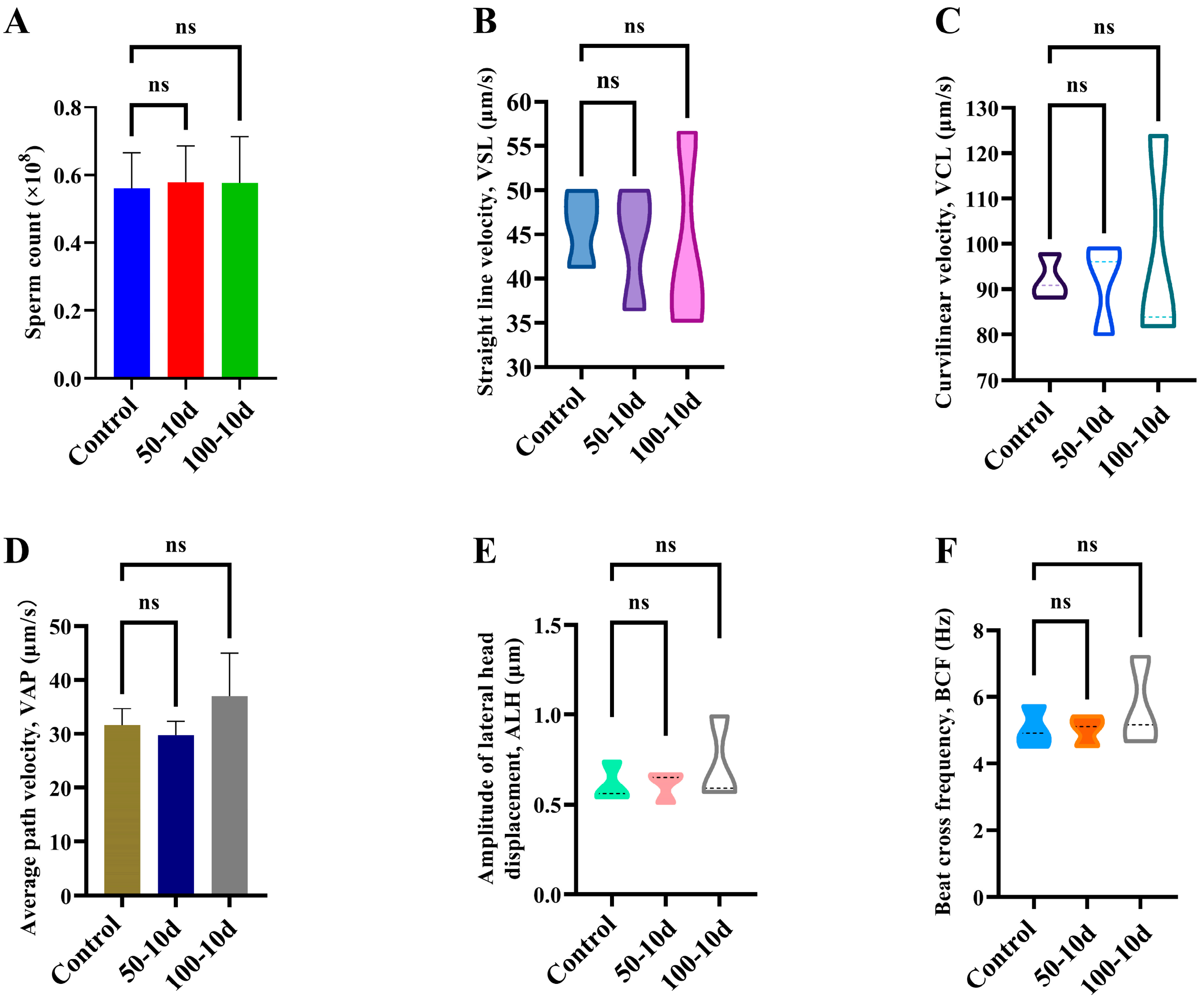

Figure S9. Sperm viability was assayed on day 10 after 100 mg/kg2-BP injection. (A) Sperm count; (B) Straight-line velocity; (C) Curvilinear velocity; (D) Average path velocity; (E) Lateral amplitude; (F) Beat frequency. Significance levels were denoted as * for $p < 0.05$, ** for $p < 0.01$, *** for $p < 0.001$ and non-significant (ns).
